# Mouse hippocampal GABAB1 but not GABAB2 subunit-containing receptor complex levels are paralleling retrieval in the multiple-T-maze

**DOI:** 10.3389/fnbeh.2015.00276

**Published:** 2015-10-19

**Authors:** Soheil K. Falsafi, Maryam Ghafari, András G. Miklósi, Ephrem Engidawork, Marion Gröger, Harald Höger, Gert Lubec

**Affiliations:** ^1^Department of Pediatrics, Medical University of ViennaVienna, Austria; ^2^Department of Pharmacology and Clinical Pharmacy, School of Pharmacy, College of Health Sciences, Addis Ababa UniversityAddis Ababa, Ethiopia; ^3^Skin and Endothelium Research Division, Department of Dermatology, Medical UniversityVienna, Austria; ^4^Core Unit of Biomedical Research, Division of Laboratory Animal Science and Genetics, Medical University of ViennaVienna, Austria

**Keywords:** spatial learning, mice, multiple T-maze, GABAB receptor, receptor complex

## Abstract

GABAB receptors are heterodimeric G-protein coupled receptors known to be involved in learning and memory. Although a role for GABAB receptors in cognitive processes is evident, there is no information on hippocampal GABAB receptor complexes in a multiple T maze (MTM) task, a robust paradigm for evaluation of spatial learning. Trained or untrained (yoked control) C57BL/6J male mice (*n* = 10/group) were subjected to the MTM task and sacrificed 6 h following their performance. Hippocampi were taken, membrane proteins extracted and run on blue native PAGE followed by immunoblotting with specific antibodies against GABAB1, GABAB1a, and GABAB2. Immunoprecipitation with subsequent mass spectrometric identification of co-precipitates was carried out to show if GABAB1 and GABAB2 as well as other interacting proteins co-precipitate. An antibody shift assay (ASA) and a proximity ligation assay (PLA) were also used to see if the two GABAB subunits are present in the receptor complex. Single bands were observed on Western blots, each representing GABAB1, GABAB1a, or GABAB2 at an apparent molecular weight of approximately 100 kDa. Subsequently, densitometric analysis revealed that levels of GABAB1 and GABAB1a but not GABAB2- containing receptor complexes were significantly higher in trained than untrained groups. Immunoprecipitation followed by mass spectrometric studies confirmed the presence of GABAB1, GABAB2, calcium calmodulin kinases I and II, GluA1 and GluA2 as constituents of the complex. ASA and PLA also showed the presence of the two subunits of GABAB receptor within the complex. It is shown that increased levels of GABAB1 subunit-containing complexes are paralleling performance in a land maze.

## Introduction

Gamma-aminobutyric acid (GABA) is the primary inhibitory neurotransmitter in the central nervous system and mediates its effect through two distinct classes of GABA receptors: the ionotropic (GABAA and GABAC) receptors, which, when activated produce a rapid and very short-lived inhibition via chloride currents (Watanabe et al., 2002) and the metabotropic GABAB receptor, which is responsible for slow but sustained inhibitory currents (Couve et al., 2000; Bettler et al., 2004).

GABAB receptors are obligatory heterodimers composed of GABAB1 and GABAB2 subunits, which are required for agonist binding and G-protein coupling (Robbins et al., 2001). The receptors are implicated in numerous behaviors and central nervous system disorders, including epilepsy, depression, anxiety, panic, and sleep disorders (Bettler et al., 2004). Molecular diversity of GABAB receptors arise from the two pharmacologically indistinguishable GABAB1 isoforms (1a and 1b) (Bettler et al., 2004; Pinard et al., 2010) and from potassium channel tetramerization domain-containing (KCTD) auxiliary subunits (Schwenk et al., 2010).

Both, GABAB1a and GABAB1b receptor subunits are expressed in the hippocampus (Liang et al., 2000), where they can modulate neuronal excitability. It is suggested that GABAB receptors containing the GABAB1a subunit are primarily located presynaptically and those with GABAB1b subunits are primarily postsynaptic (Vigot et al., 2006). However, this distribution may vary with the brain region and whether the presynaptic neuron is GABAergic or glutamatergic (Waldmeier et al., 2008). Heteroreceptors are predominantly found at glutamatergic boutons, mediating presynaptic inhibition of glutamate release largely via suppression of Ca^2+^ currents (Bussières and El Manira, 1999; Barral et al., 2000; Vigot et al., 2006; Waldmeier et al., 2008), while postsynaptic receptors activate slow inhibitory postsynaptic potentials through activation of inwardly rectifying K^+^ channels (Fernández-Alacid et al., 2009; Liu et al., 2012). In addition, GABAB receptors can function as autoreceptors in interneurons (Waldmeier et al., 2008) and also modulate adenylyl cyclase, through which they influence downstream molecular pathways (Couve et al., 2000; Bettler et al., 2004).

A substantial body of evidence exists indicating the modulatory role of GABAB receptors in learning and memory. Stimulation of GABAB receptors impaired working memory in the basal forebrain (DeSousa et al., 1994) and medial septum (Stackman and Walsh, 1994) using double Y- and radial maze. Conversely, a series of centrally active GABAB receptor blockers have been shown to improve performance in a host of learning paradigms, including passive avoidance (Bianchi and Panerai, 1993; Mondadori et al., 1993), active avoidance (Getova and Bowery, 1998), and radial maze (Helm et al., 2005). However, Brucato et al. (1996) reported that the antagonist CGP 46381 showed no effect in radial maze and decreased performance in a water maze (Brucato et al., 1996).

Crosstalk among the neurotransmitter repertoire is common in the central nervous system. This is made possible through formation of signaling complexes as a result of receptor assembly with other receptors or regulatory proteins. As studies so far have not been addressing GABAB receptor containing complexes as actors in the MTM, it was the aim of the current study to show which GABAB-containing receptor complex(es) are involved in a MTM task, a paradigm for evaluation of spatial learning.

## Experimental procedures

### Animals

C57BL/6J (*n* = 20, male, aged 10–12 weeks, 10 Trained which are quick learners and were selected from 20 animals and 10 Untrained) mice were used for the study. C57BL/6J mice were obtained from JANVIER SAS laboratory (France). All mice were kept and maintained in polycarbonate cages Type II (207 × 140 × 265 mm, Ehret, Austria) filled with autoclaved wood chips (Ligncell select, Rettenmaier, Austria) in the core unit of Biomedical Research, Division of Laboratory Animal Science and Genetics, Medical University of Vienna. The animals were housed in groups with access to autoclaved Altromin standard rodent diet (Altromin, Germany) and water *ad libitum*, and maintained under standard conditions (ambient temperature of 22 ± 1°C, relative humidity of 50 ± 10%, light/dark cycle of 14:10, and ventilation with 100% fresh air that resulted in an air change rate of 15 times per hour). The room was illuminated with artificial light at an intensity of about 200 l × in 2 m from 5 a.m. to 7 p.m. The experiments were carried out under a license obtained from the Federal Ministry of Education, Science and Culture, which includes an ethical evaluation of the project (Project: BMWF-66.009/0240-II/10b/2009). Housing and maintenance of animals were in compliance with European and national regulations (Ghafari et al., 2012a).

### Multiple-T-maze

MTM is one of the spatial learning tasks in which animals learn to find the goal box based on their memory of previously visited arms and carried out as described elsewhere (Patil et al., 2009), with minor modifications. The MTM was constructed of wood and consisted of a platform with seven choice points, and dimensions of 150 × 130 × 15 cm and a path width of 8 cm. Before testing, mice were deprived of food for 16 h to motivate food searching. Ten mice were then placed in a start box (diameter: 10.5 cm) and allowed to search the reward. The trial was considered to be completed when the mice either reached the goal box or failed to find it within 5 min. In the goal box, mice were allowed to consume a small piece of food pellet provided as a reward. Immediately after each trial, the entire maze was cleaned with 1% incidin solution (Incidin extraN, Lohmann and Rauscher, Austria) to remove possible cues. Another 10 mice were used as untrained controls (yoked controls) that spent the same time in the MTM without learning performance as there was no reward in the goal box. After testing, animals were returned to their cage and given food based on their body weight (120 g/kg) so as to preserve their weight and keep them starved for the following day task. Mice were trained for 4 days, with three trials per day having 20 min interval between each trial. Trials were recorded using computerized tracking/image analyzer system (video camcorder: 1/3 in. SSAMHR EX VIEW HAD coupled to the computational tracking system: TiBe-Split). The system provides the following parameters: correct or wrong decision, path length, speed, and latency to reach the goal box. Six hours following the last training on day 4, animals were euthanized by neck dislocation. Hippocampi were exstirpated from the brain within 1 min and kept at –80°C until analyses (Falsafi et al., 2012).

### Sample preparation

A total of 20 samples (obtained from 10 trained and 10 untrained mice) were homogenized in ice-cold homogenization buffer (10 mM HEPES, pH 7.5, 300 mM sucrose and 1 complete protease inhibitor tablet [Roche Molecular Biochemicals, Mannheim, Germany] per 50 mL) using an Ultra-Turrax homogenizer (IKA, Staufen, Germany). The homogenate was then centrifuged for 10 min at 1000 × g, and the pellet was discarded. The supernatant was subsequently centrifuged at 50,000 × g for 30 min in an ultracentrifuge (Beckman Coulter Optima L-90K), and the resulting pellet was homogenized in 5 mL washing buffer (homogenization buffer without sucrose), incubated on ice for 30 min and centrifuged at 50,000 × g for an additional 30 min (Falsafi et al., 2012).

### Sucrose gradient ultracentrifugation

The plasma membrane purification procedures for the obtained pellets were performed as previously described with slight modifications (Ghafari et al., 2012a). Sucrose density gradients were formed by carefully layering solutions of 700 μL of each of 69, 54, 45, 41, and 37% (w/v) on top of each other. Membrane pellets were re-suspended in the homogenizing buffer, and 500 μL were layered on top of the tubes that were then finally filled with the homogenizing buffer. Samples were ultracentrifuged at 4°C and 70,000 × g for 3 h. After centrifugation, the 41% fraction was collected from the sucrose interface, diluted 10 times with the homogenizing buffer, and then ultracentrifuged at 4°C and 100,000 × g for 30 min. After discarding the supernatant, the pellet was stored at −80°C until use.

### Blue native-polyacrylamide gel electrophoresis (BN-PAGE)

Membrane pellets obtained from the 41% fraction of sucrose gradient ultracentrifugation were solubilized in extraction buffer [1.5 M 6-aminocaproic acid, 300 mM Bis–Tris, pH 7.0] and 10% Triton X-100 stock solution was added, at a ratio of 1:4, to achieve a final concentration of 2% Triton X-100, with vortexing every 10 min for 1 h. Following solubilization, samples were cleared by centrifugation at 4°C and 20,000 × g for 60 min. The protein content was estimated using the BCA protein assay kit (Pierce, Rockford, IL, USA).

Following mixing of 100 μL of the membrane protein preparation with 16 μL BN-PAGE loading buffer [5% (w/v) Coomassie G250 in 750 mM 6-aminocaproic acid], 50 μg of the membrane protein preparations were loaded onto gels. BN-PAGE was performed in a PROTEAN II xi Cell (BioRad, Germany) using 4% stacking and 5–18% separating gels. The BN-PAGE gel buffer contained 500 mM 6-aminocaproic acid, 50 mM Bis–Tris, pH 7.0; the cathode buffer contained 50 mM Tricine, 15 mM Bis–Tris, 0.05% (w/v) Coomassie G250, pH 7.0; and the anode buffer contained 50 mM Bis–Tris, pH 7.0. The voltage was set to 50 V for 1 h followed by 75 V for 6 h, and then sequentially increased to 400 V (maximum current 15 mA/gel, maximum voltage 500 V) until the dye front reached to the bottom of the gel (Ghafari et al., 2012a). Native high molecular mass markers were obtained from Invitrogen (Carlsbad, CA, USA).

### Immunoblotting

Membrane proteins were transferred from BN-PAGE to PVDF membranes. After blocking of the membranes for 1 h with 10% non-fat dry milk in 0.1% TBST (100 mM Tris–HCL, 150 mM NaCl, pH 7.5, 0.1% Tween 20), mouse anti-GABAB1 (Abcam, ab55051, Cambridge, UK; 1/5000). Goat anti-GABAB1a (sc-73401/2500), goat anti-MuscarinicM2 (Abcam, ab140473, Cambridge, UK; 1/3000) and rabbit anti-GABAB2 (Abcam, ab75838, Cambridge, UK; 1/5000). The antibodies were detected using horseradish peroxidase-conjugated secondary anti-rabbit IgG antibodies (Abcam, ab6721, Cambridge, UK; 1/10,000), anti-Goat IgG antibodies (Abcam, ab6741, Cambridge, UK; 1/5000), Anti-Mouse IgG antibodies (Abcam, 97035, Cambridge, UK; 1/10,000) Membranes were then developed with the ECL Plus Western Blotting Detection System (GE Healthcare, Buckinghamshire, UK). All membranes were stained with Coomassie blue R-350 (GE Healthcare, Buckinghamshire, UK, Falsafi et al., 2012). Arbitrary optical densities of immunoreactive bands were measured by the Image J software program (http://rsb.info.nih.gov/ij/). Loading controls for the BN-PAGE western blot were carried out according to Welinder and Ekblad (Welinder and Ekblad, 2011).

### Immunoprecipitation of the GABAB1 receptor

Total hippocampal membrane fractions were suspended in lysis buffer containing 1% Triton X100, 150 mM NaCl, 1 mM EDTA, 50 mM Tris-HCl (pH 8.0), 10 mM NaF, 10 mM Na3VO4 and protease inhibitor cocktail (Roche, Mannheim, Germany) on a rotating shaker for 1 h at 4°C. After centrifugation at 15,300 × g and 4°C for 10 min, the supernatant was incubated with affinity purified rabbit antibody against GABAB1 and subsequently incubated with protein G agarose beads (GE Healthcare, Uppsala, Sweden) for 4 h at 4°C with gentle rotation. After washing five times with the same lysis buffer, proteins bound were denatured with sample buffer containing 125 mM Tris (pH 6.8), 4% SDS, 20% glycerol, 10% beta-mercaptoethanol, and 0.02% bromophenol blue at 95°C for 3 min. Samples were then loaded onto 10% SDS-polyacrylamide gels, electrophoresed and subsequently transferred to PVDF membranes and analyzed by the western blotting as described in the previous subsection (Ghafari et al., 2012b).

### In-gel digestion of proteins and peptides

Bands that were recognized by the corresponding antibodies against the GABAB1 receptor subunit were picked from the SDS gel and put into a 1.5 mL tube. Gel pieces were initially washed with 50 mM ammonium bicarbonate and then two times with washing buffer (50% 100 mM ammonium bicarbonate/50% acetonitrile) each time for 30 min with vortexing. An aliquot of 100 μL of 100% acetonitrile was added to the tubes to cover the gel pieces completely and the mixture was incubated for 10 min. Gel pieces were dried using a SpeedVac concentrator. Reduction of cysteine residues was carried out with 10 mM dithiothreitol (DTT) solution in 100 mM ammonium bicarbonate (pH 8.6) for 60 min at 56°C. After discarding the DTT solution, the same volume of 55 mM iodoacetamide (IAA) solution in 100 mM ammonium bicarbonate buffer (pH 8.6) was added and incubated in darkness for 45 min at 25°C to achieve alkylation of cysteine residues. The IAA solution was replaced by washing buffer (50% 100 mM ammonium bicarbonate/50% acetonitrile) and washed twice for 15 min each time with vortexing. Gel pieces were washed and dried in 100% acetonitrile followed by dryness in a SpeedVac. Dried gel pieces were re-swollen with 12.5 ng/μL trypsin (Promega, Germany) solution reconstituted with 25 mM ammonium bicarbonate. Gel pieces were incubated for 16 h (overnight) at 37°C (trypsin). The supernatant was transferred to new 0.5 mL tubes, and peptides were extracted with 50 μL of 0.5% formic acid / 20% acetonitrile for 20 min in a sonication bath. This step was repeated two times. Samples in extraction buffer were pooled in 0.5 mL tubes and evaporated in a SpeedVac concentrator. The volume was reduced to approximately 20 μL and an equal volume of HPLC grade water (Sigma, Germany) was then added for spectrometric analysis (Ghafari et al., 2012b).

### Nano-LC–ESI–CID/ETD-MS/MS

The analysis utilized an Ultimate 3000 HPLC system (Dionex, Sunnyvale, CA) equipped with a PepMap100 C-18 trap column (300 μm × 5 mm) and a PepMap100 C-18 analytic column (75 μm × 150 mm). The gradient applied was as follows: A = 0.1% FA in water and B = 0.08% FA in acetonitrile: 4–30% B from 0 to 105 min, 80% B from 105 to 110 min, and 4% B from 110 to 125 min. An HCT ultra ETD II (Bruker Daltonics, Bremen, Germany) was used to record the peptide spectra over a mass range of m/z 350–1500 and MS/MS spectra via information-dependent data acquisition over a mass range of m/z 100–2800. The MS spectra were repeatedly recorded, followed by four data-dependent CID MS/MS spectra and four ETD MS/MS spectra generated from the four highest intensity precursor ions. Active exclusion of 0.4 min following 2 spectra was used to detect low-abundance peptides. The voltage between the ion spray tip and spray shield was set to 1500 V. Drying nitrogen gas was heated to 150°C, and the flow rate was set to 10 L/min. The collision energy was set automatically according to the mass and the charge state of the peptides chosen for fragmentation. Multiple charged peptides were chosen for the MS/MS experiments due to their good fragmentation characteristics. The obtained MS/MS spectra were interpreted, and peak lists were generated with DataAnalysis 4.0 (Bruker Daltonics, Bremen, Germany).

MASCOT searches against the most recent version of the UniProtKB database were performed using MASCOT 2.2.06 (Matrix Science, London, UK) for protein identification. The search parameters were set as follows: enzymes selected as trypsin with a maximum of 2 missing cleavage sites; species taxonomy limited to mouse; a mass tolerance of 0.2 Da for peptide tolerance, and 0.2 Da for MS/MS tolerance; an ion score cutoff below 15; fixed modification of carbamidomethyl (C); and variable modification of oxidation (M), deamidation (N, Q), and phosphorylation (S, T, Y). Proteins were positively identified based on a significant MOWSE score. Following protein identification, an error-tolerant search was performed to detect non-specific cleavage and unassigned modifications. The returned protein identification information was manually inspected and filtered to confirm the protein identification lists.

Higher sequence coverage was obtained using Modiro® software with the following parameters: the selected enzymes exhibited two maximum missing cleavage sites; a peptide mass tolerance of 0.2 Da was selected for peptide tolerance; 0.2 Da was used as the fragment mass tolerance; and modification 1 of carbamidomethyl (C) and modification 2 of methionine oxidation were applied. Positive protein identification was first based on the obtained spectra, and each identified peptide was subsequently considered significant based on its ion charge status, b- and y-ion fragmentation quality, ion score (>200) and significance scores (>80). The protein identifications were manually inspected and filtered to obtain the confirmed lists of identified proteins (Ghafari et al., 2012b).

### Antibody shift assay

For the antibody shift assay (ASA), samples were pre-incubated for an hour with mouse anti-GABAB1 (Abcam, ab55051, Cambridge, UK; 1/5000) and rabbit anti-GABAB2 (Abcam, ab75838, Cambridge, UK; 1/5000), with gentle shaking at 4°C, followed by loading the mixture onto the BN gel. The lanes from the BN gel were cut out and the gel strips were equilibrated for 30 min in equilibration buffer [1% (w/v) SDS and 1% (v/v) 2-mercaptoethanol] with gentle shaking and then briefly rinsed twice with SDS-PAGE electrophoresis buffer [25 mM Tris–HCl, 192 mM glycine and 0.1% (w/v) SDS; pH 8.3] and subsequently by Milli-Q water. SDS-PAGE was performed in a PROTEAN II xi Cell using 4% stacking gel and 10% separating gel. The gel strips were run at 12°C with an initial current of 50 V (during the 1st h). The voltage was subsequently increased to 100 V for the next 12 h (overnight) and then increased to 150 V until the dye front reached the bottom of the gel. The gel was then further subjected to a western blotting analysis as described earlier.

### Proximity ligation assay

Three C57BL/6J mice were anesthetized with 0.3 ml/kg intraperitoneal injection of sodium penthobarbital (Release, 300 mg/ml, Wirtschaftsgenossenschaft deutscher Tierärzte eG, Grabsen, Germany) and perfused intracardially with ice-cold PBS (0.1M phosphate buffered saline, pH 7.2), containing 0.2% heparin followed by 4% paraformaldehyde at a pH of 7.4 in PBS. Brain samples were post-fixed in 4% paraformaldehyde for 24 h at 4°C then transferred into a 30% sucrose solution (in PBS) for 48 h. Brain tissues were embedded with Tissue-Tek media (OCT compound, Sakura Finetek Europe, The Netherlands) then immersed in isopentane cooled with dry ice and sectioned at 30 μm with a cryostat (Leica CM 3050S, Wetzlar, Germany).

The Proximity Ligation Assay (PLA) was performed according to the manufacturer's protocol (O-LINK Bioscience, Uppsala, Sweden) with slight modifications. Free floating brain slices were blocked for 30 min at room temperature using blocking buffer supplied with the kit. After blocking, brain slices were incubated with diluted mouse monoclonal anti-GABAB1 (1:50, Abcam, Cambridge, UK) and rabbit polyclonal anti-GABAB2 receptor (1:25 Abcam, Cambridge, UK) primary antibodies for 72 h at 4°C on a rocking platform. Following incubation with primary antibodies, slices were washed with wash buffer A (O-LINK Bioscience, Uppsala, Sweden) and then incubated with rabbit PLUS and mouse MINUS probes (1:40, O-LINK Bioscience, Uppsala, Sweden) for 2 h at 37°C with gentle orbital shaking. Ligation was performed according to the manufacturer's protocol with the exception of the incubation time (45 min were used instead of 30 min mentioned in the protocol). DNA polymerase was diluted (1:20) and incubated for 120 min at 37°C. The rest of the amplification steps remained unchanged. After amplification, slices were washed with 1 × then 0.01 × wash buffer B prepared according to the recipe supplied in the kit manual. Finally, brain slices were transferred to glass slides, mounted with Duolink *in Situ* Mounting Medium with DAPI (O-LINK Bioscience, Uppsala, Sweden). Images were acquired with a Zeiss LSM 780 confocal laser scanning microscope (Carl Zeiss GmbH, Jena, Germany) at 100 × magnification keeping all acquisition settings even through all samples.

### Statistical analyses

Data obtained from MTM and western blotting are presented as mean ± SD. Data from western blot were analyzed by unpaired Student's *t*-test, while those of MTM were analyzed using ANOVA with repeated measure. Statistical analysis and calculations were performed using SPSS for Windows 19.0 and GraphPad Prism (San Diego, CA, USA).

## Results

### Behavioral studies

**Figure 1:**
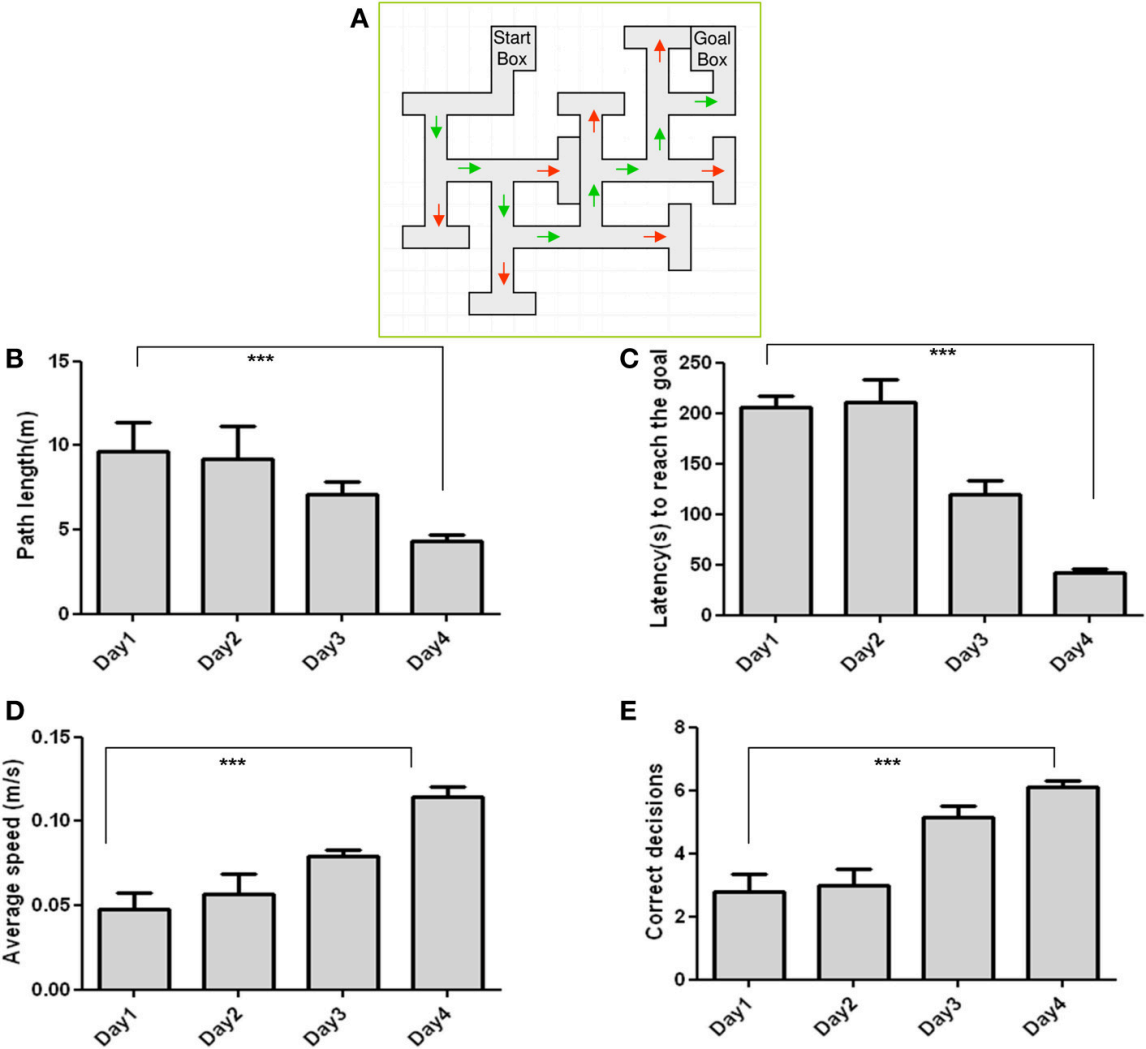
**Performance of C57BL/6J in the Multiple-T maze task: (A) Measured parameters include Path length (B), Latency (C), Speed (D), and Correct decisions (E)**. Latency and path length decreased, while speed and correct decision increased across the training days. *n* = 10; Data are expressed as mean ± SD and were analyzed by ANOVA repeated measures; ^***^p < 0.001.

Mice learned the maze quickly and efficiently Schematic of the Multiple T maze (MTM) (Figure 1A). Training enabled the animals to decrease their path length (Figure 1B) across training days. Latency was relatively unchanged in the first 2 days of training but was reduced remarkably thereafter (Figure 1C). Animals also refined their path with each training day as demonstrated by an increase in the average speed to reach the goal box (Figure 1D). Errors appear to be left for chance in the first 2 days of the training but started to decrease thereafter, as revealed by a significantly increased number of correct decisions on day 4 (Figure 1E).

### Protein-based studies

**Figure 2:**
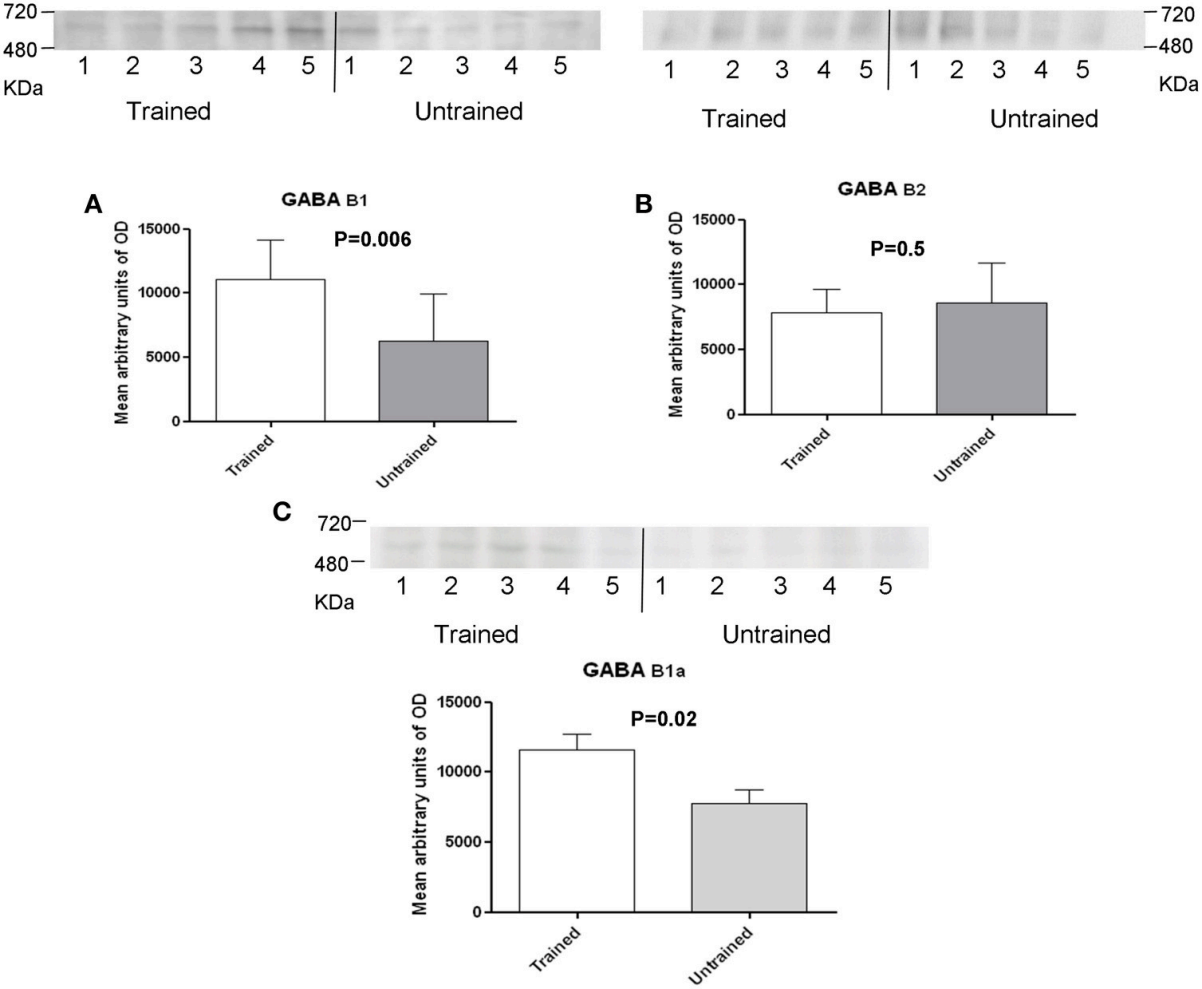
**Western blot analysis of training-induced changes in levels of GABAB1 and GABAB1a (A,C) and GABAB2 (B) subunits containing receptor complex in C57BL/6J mice, the number of mice are shown in each bar (A) there were samples from 9 untrained and 10 trained animals: membrane preparations obtained as described in the method section were subjected to BN-SDS-PAGE followed by Western blotting**. Subunits migrated in a blue native gel as a GABAB receptor complex at anapparent molecular weight of 500 and 600 kDa (Range, 480–720 kDa). Levels were determined measuring arbitrary optical densities of immunoreactive bands using the Image G software; data are expressed as mean ± SD and analyzed by unpaired student's *t*-test; ^*^p < 0.01 compared to untrained mice.

**Figure 3:**
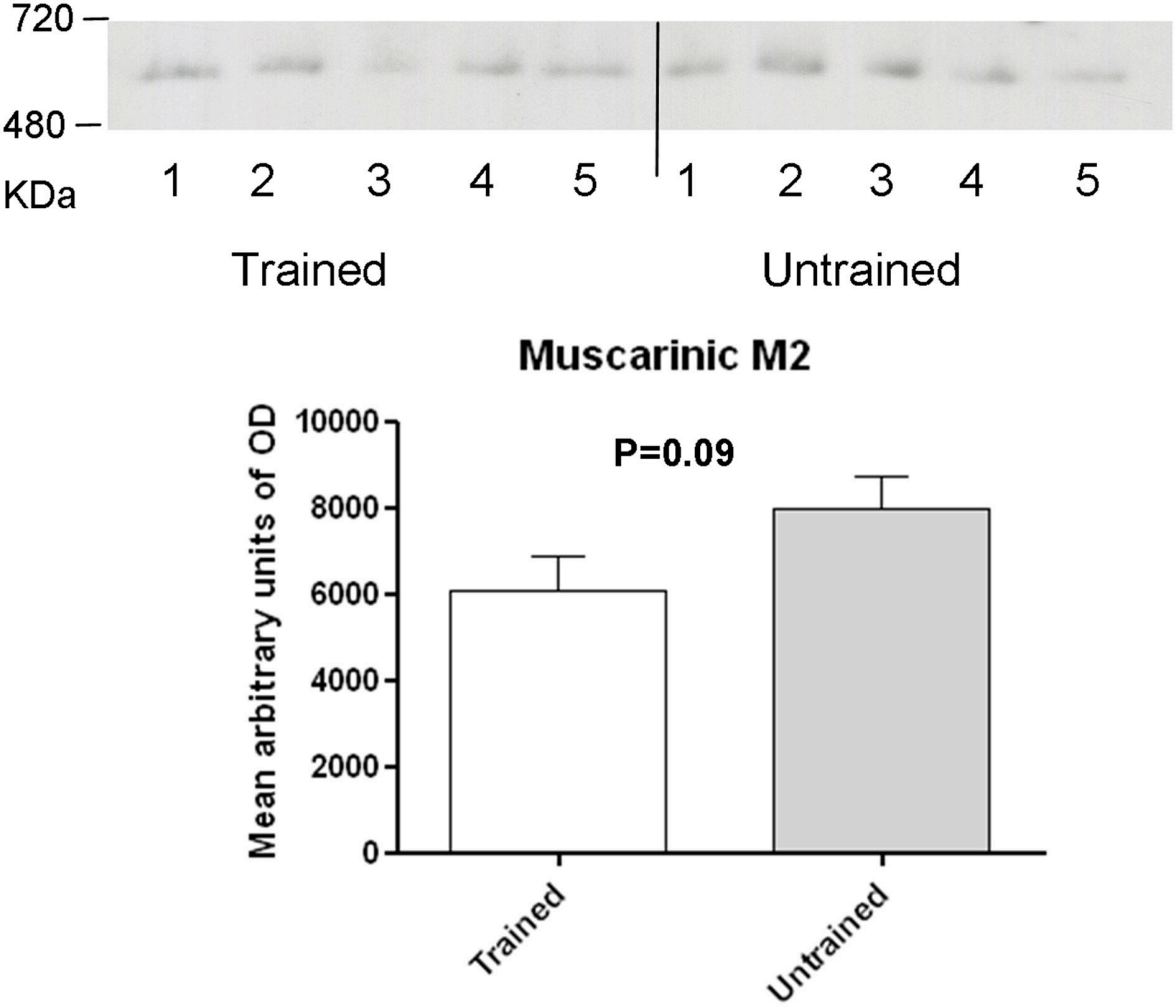
**Western blot analysis of training-induced changes in levels of muscarinic type 2 receptor containing complex in C57BL/6J mice: membrane preparations obtained as described in the method section were subjected to BN-PAGE followed by western blotting**. Complexes migrated in a blue native gel as a muscarinic type 2 containing receptor complex at an apparent molecular weight of 600 kDa (Range, 480–720 kDa). Levels were determined measuring arbitrary optical densities of immunoreactive bands using the Image G software. Data are expressed as mean ± SD and analyzed by student's *t*-test.

To assess the role of GABAB receptors in the MTM task, exstirpated hippocampi from trained and untrained mice were subjected to blue native-PAGE followed by western blotting (BN-PAGE-WB). The analysis revealed the presence of a complex containing GABAB1 and GABAB2 subunits at an apparent molecular weight of approximately 600 kDa (Figure 2A) and 500 kDa (Figure 2B), (range 480–720 kDa). Subsequent quantitative analysis was made to see the effect of training on receptor levels. It was found out that a complex containing the GABAB1 (Figure 2A), and GABAB1a (Figure 2C), subunit containing receptor complex levels were significantly increased (*p* < 0.01) in trained compared to untrained mice. By contrast, there was no apparent difference between the two groups when GABAB2 subunit containing receptor complex levels were considered (Figure 2B). In addition, based on reports demonstrating direct interaction between GABAB2 and muscarinic subtype 2 (M2) receptors through receptor assembly at the plasma membrane (Boyer et al., 2009), an attempt was also made to detect and quantify M2 containing receptor complex levels using BN-PAGE-WB. M2 was detected as having an apparent molecular weight of approximately 600 kDa, but its levels did not exhibit any significant difference between trained and untrained animals (Figure 3).

Assays used for analysis of proteins showed the presence of the two subunits within the complex. The ASA is often used to verify the co-existence of a protein in a complex. The binding of specific antibodies to a target protein causes a shift of the whole target protein complex on the BN-PAGE by the added mass of the antibody. A corresponding mass shift of a protein complex, therefore, is indicative of the presence of the target protein in the complex. In this assay, antibodies specific for B1 and B2 subunits shifted the complex to higher apparent molecular weights, with the discrete increments most likely reflecting assembly of these subunits into GABAB receptors.

**Figure 4:**
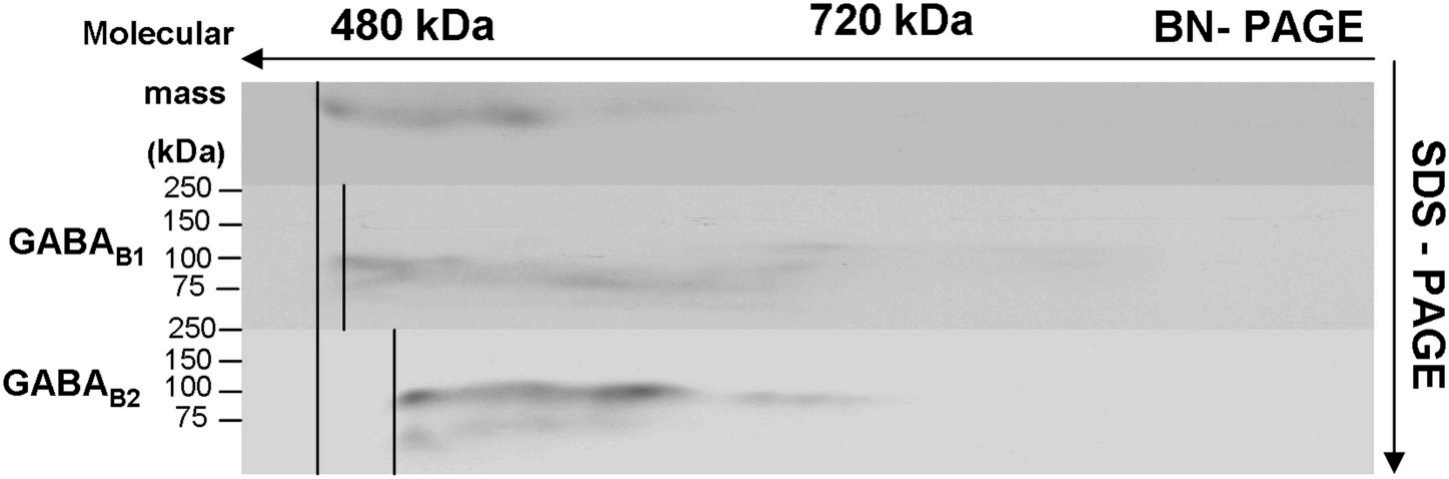
**Antibody shift assay to show the presence of GABAB subunits in receptor complexes: membrane preparations were incubated with and without an antibody against the respective subunits of GABAB1 and GABAB2 and subjected to BN-SDS-PAGE**. Complexes incubated with the respective antibody shifted toward the high molecular weight (middle and lower bands) compared to controls (upper band).

ASA was performed to provide additional evidence for co-assembly of the two subunits in order to form functional receptors (Figure 4). The assay revealed that the band in the control (without incubation of an antibody) (upper band) was shifted toward a high molecular weight when incubated with anti-GABAB1 antibody (central band) and with anti-GABAB2 antibody (lower band).

**Figure 5:**
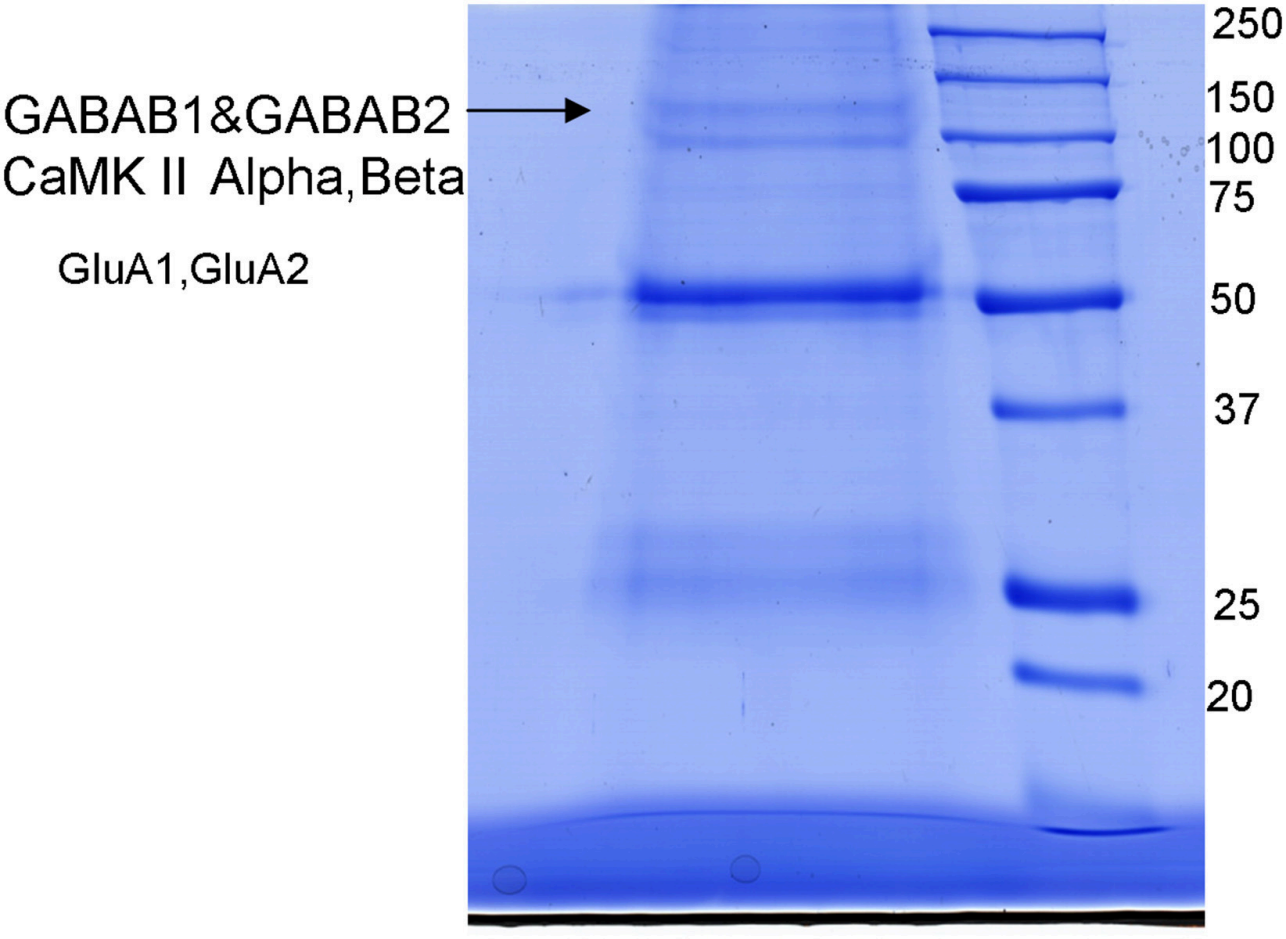
**Immunoprecipitation using an antibody against the GABABl subunit run on an SDS-PAGE**. The resulting gel showed the pattern of probable receptor complex composition containing GABAB1, GABAB2, Calcium/calmodulin-dependent protein kinase [CaMKII α and β isoforms, Glutamate receptor type1 (GluA1), and 2 (GluA2)]. Mass spectrometry was carried out for identification of receptor proteins picking the bands shown by arrows.

Immunoprecipitation was carried out using an antibody against the GABAB1 subunit (also used for Western blotting, see above) and the subunit was shown to co-precipitate with GABAB2. the GABAB1 receptor subunit was detected at an apparent molecular weight of approximately 100 kDa (Figure 5). Mass spectrometry following in-gel digestion of bands from the corresponding SDS-PAGE showed the presence of GABAB1, GABAB2, Calcium Calmodulin kinase II (CaMKII α and β subunits), Glutamate receptor 1 (GluA1), and Glutamate receptor 2 (GluA2). Data from mass spectrometry indicating the total sequence coverage for the individual proteins in the immunoprecipitate are provided in Table 1. Peptides Peptides used for the protein identifications are given in the Supplementary Table 1.

**Table 1:** **Mass spectrometric identification of the GABAB1 subunit containing receptor complex**.

**Receptor**	**Accession number**	**Length**	**Mass (Da)**	**Sequence coverage (%)**
GABAB1	Q9WV18	960	108	23
GABAB2	Q80T41	940	105	17
CaMKII Alpha	P11798	478	54	21
CaMKII Beta	P28652	542	60	12
GRIA1	P23818	907	101	3
GRIA2	P23819	883	98	10

*Bands picked from SDS-PAGE and subjected to MS/MS analysis*.

### PLA

**Figure 6:**
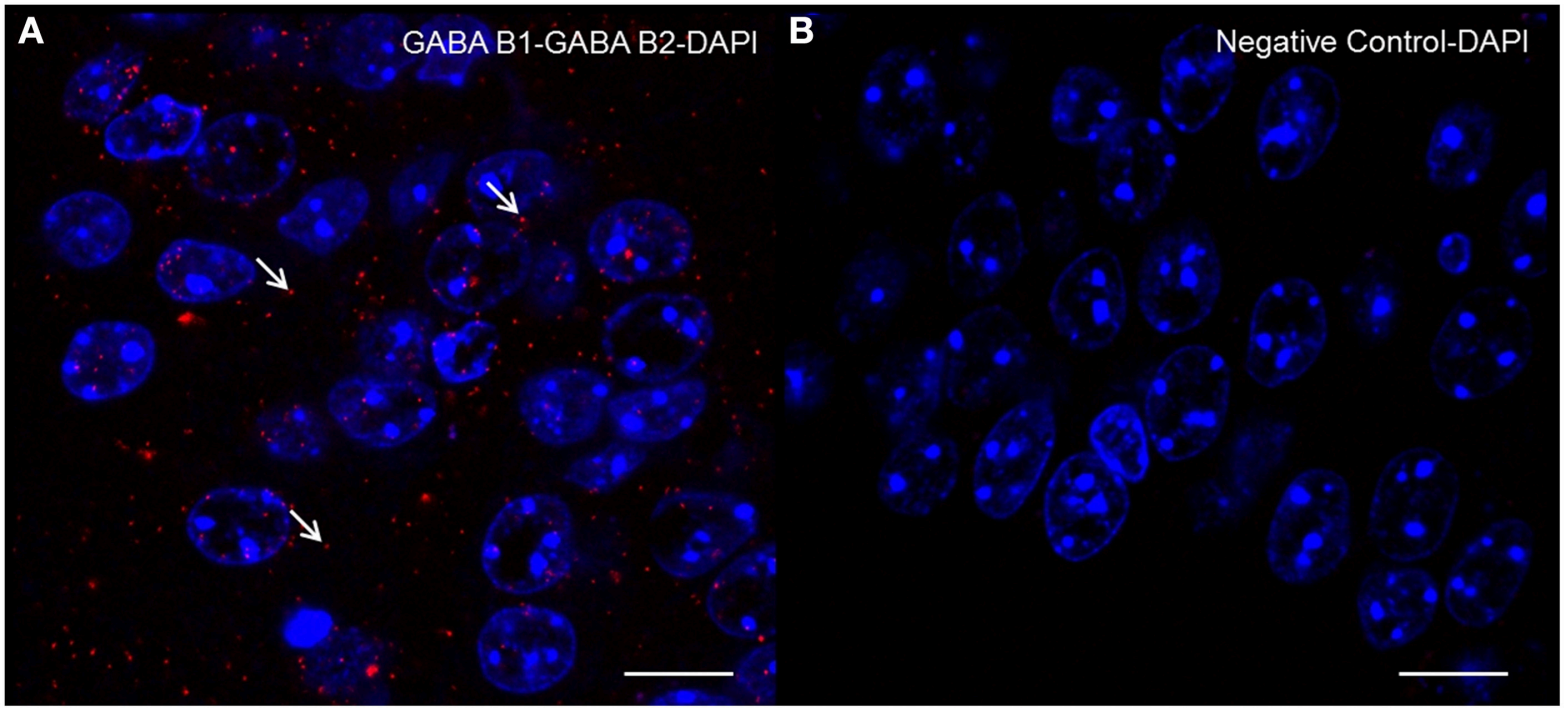
**Detection of GABAB1-GABAB2 receptor complexes by “***in situ***” Proximity Ligation Assay: Brain slices were incubated with primary antibodies for the two subunits**. Slices were then incubated with plus and minus probes, ligated, and amplified. After that, slices were mounted and images were acquired through confocal microscopy. Representative images showing the GABAB1-GABAB2 receptor complex **(A)** negative control with no primary antibody **(B)** counterstained nuclei with DAPI in mouse hippocampus. Each signal represents the proximity/probable interaction of GABAB1 with GABAB2 receptors. The arrows indicate examples of signals. Confocal microscope images acquired at 100× magnification. Scale bar 10 μm.

Evidence for the presence of the two GABAB subunits within the complex was provided by the PLA (Figure 6). The assay produced a typical number of discrete fluorescent spots, demonstrating the co-localization of both GABAB1 and GABAB2 subunits within the complex in the hippocampal formation.

## Discussion

Spatial learning and memory is largely dependent on the hippocampus and is mediated by persistent changes in neuronal structure and activity. GABAB receptors are localized in the perisynaptic areas of dendritic spines as well as in the dendritic shafts, which enable them to regulate synaptic inputs (Bettler and Tiao, 2006). As a result, changes in the activity of GABAB receptors are critical for controlling neuronal activity and cognitive functioning (Bowery et al., 2002).

The use of BN-PAGE allows subsequent immunoblotting of strongly hydrophobic membrane proteins as receptors and native receptor complexes including high molecular weight material (Kang et al., 2009). The GABAB1 and GABAB2 complexes could therefore be detected in their native forms. The present study alluded to the fact that trained mice displayed an enhanced performance in MTM, a spatial learning task, (Figure 1) and this performance was paralleled by an increased expression of a GABAB1/1a containing receptor complex.

Most of the evidence relating GABAB receptors to learning and memory came out of studies using pharmacological ligands. Accordingly, antagonists appear to enhance cognition in majority of the cases (Bianchi and Panerai, 1993; Mondadori et al., 1993; Staubli et al., 1999; Helm et al., 2005), but have either neutral or inhibitory effect in a few cases (Brucato et al., 1996). The pattern for agonists is variable, ranging from impairment (DeSousa et al., 1994; Nakagawa and Takashima, 1997; Cryan et al., 2004; Zarrindast et al., 2004) through facilitation (Georgiev et al., 1988; Saha et al., 1993) to no effect (Cryan et al., 2004; Li et al., 2013). Possible explanations for these discrepancies could be that: (i) behavioral paradigms that assess learning and memory differentially involve stress- or anxiety-related behavior; therefore, the underlying neurobiology and pharmacology are not identical (Sharma et al., 2010) large species and strain differences exist in these paradigms (Sharma et al., 2010) dose administered (Li et al., 2013) and outcomes assessed (learning or retention) (Cryan et al., 2004) could be different: or (iv) as selectivity of these ligands for the two isoforms of GABAB1 is unclear, they can differentially bind to one of the two isoforms, thereby producing different effects. As no antibody against GABAB1b was available, herein the GABAB1a isoform was determined which was significantly increased in the GABAB1-containing complex paralleling retrieval in the MTM.

Subunit phosphorylation appears to regulate the surface dynamics of GABAB receptors. Phosphorylation at S867 by calcium/calmodulin-dependent protein kinase II (CaMKII) triggers endocytosis (Guetg et al., 2010), whereas protein kinase A (PKA) mediated phosphorylation at S892 stabilizes the receptor at the cell surface (Couve et al., 2002). The degree of activation of NMDA receptors, however, appears to control phosphorylation status of S783. Whilst transient NMDA activation leads to phosphorylation by AMP kinase and stabilization of the receptor, prolonged stimulation causes dephosphorylation by PP2A, which serves as a signal to promote degradation (Terunuma et al., 2010, 2014). Phosphorylation of S867 by CaMKII is detectable only in the GABAB1b subunit (Guetg et al., 2010), which mostly resides in the dendrites, in contrast to the GABAB1a subunit, which efficiently penetrates spines (Vigot et al., 2006). In addition, S783A mutation increased the expression of postsynaptic GABAB receptors by blocking receptor degradation, and this was accompanied by an increase in excitatory synapses and elevated surface AMPA receptors (Terunuma et al., 2014). And indeed, CaMKII and AMPA receptors GluA1 and GluA2 were co-immunoprecipitating with GABAB1.

Mice with S783A mutation displayed significant alterations in contextual fear learning and reduced long-term spatial memory in Barnes's maze, effects thought to result from enhanced expression of postsynaptic GABAB receptors (Terunuma et al., 2014). In the hippocampus, GABAB1a/2 receptors are located mainly presynaptically, whereas GABAB1b/GABAB2 receptors are located primarily postsynaptically (Vigot et al., 2006), and CGP 35348, a GABAB receptor antagonist with significantly higher affinity for post- vs. presynaptic receptors, is shown to enhance cognition (Olpe et al., 1993; Staubli et al., 1999). In the present study, an increase in GABAB1 containing, but not GABAB2 containing receptor complexes and this finding was paralleling performance in the MTM task. Findings from the current study suggest that the increased GABAB1a isoform-containing subunit involves a presynaptic mechanism. Since increased complex levels were noted following training of mice in an MTM task, the observation is consistent with the physiological role of GABAB1a containing receptors in ensuring that plasticity processes are maintained in the dynamic range (Gassmann and Bettler, 2012). Training did not have any appreciable effect on levels of GABAB2 containing receptor complexes as well as other receptors that interact with this subunit such as the M2 receptors. This suggests that although the GABAB2 subunit of the complex is required for G-protein coupling, receptor-mediated effects are largely dependent on GABAB1 subunit of the complex.

The maximal distance between the secondary antibodies in PLA is slightly larger than the distance between flurophores in energy transfer technologies, allowing validation of the molecular proximity of endogenous proteins *in vitro* or *ex vivo* (Schwenk et al., 2009; Navarro et al., 2013). The PLA produced a signal representing co-localization of the two subunits within the hippocampus providing further evidence for the presence of both subunits in a complex.

It is known that the GABAB receptor is a heterodimer receptor, but so far mass spectrometric evidence for a complex containing the subunits from mouse brain is missing. Immunoprecipitation followed by mass spectrometry produced evidence for the presence of a protein complex containing both subunits along with proteins involved in the glutamatergic signaling system. Methodologically, nano-LC–ESI–MS/MS was applied for identification of receptor subunits and unambiguous identification with high sequence coverage was obtained. This was achieved through in-gel digestion with trypsin and the use of two mass spectrometric principles, the combination of CID and ETD.

The co-precipitation of the GABAB1 receptor with proteins involved in glutamatergic signaling indicates that the two systems interact with each other through formation of a signaling complex. Indeed, previous co-immunoprecipitation followed by pull down assay showed that CaMKII associates with the GABAB1 receptor in mouse brain. This association may enable CaMKII to phosphorylate GABAB receptor at S867 and trigger endocytosis (Guetg et al., 2010). This observation is consistent with our finding of detecting CaMKII within the receptor complex.

Taken together, data presented herein demonstrate that training leads to increased levels of GABAB1/1a—containing receptor complexes but not GABAB2—containing receptor complexes in mouse hippocampus. The presence of these subunits in the complex is unequivocally confirmed using several biochemical methods including BN-PAGE, ASA, PLA, and immunoprecipitation followed by mass spectrometry. The findings are relevant for previous studies and the design of future studies on GABAB-receptors and spatial memory.

### Conflict of interest statement

The authors declare that the research was conducted in the absence of any commercial or financial relationships that could be construed as a potential conflict of interest.
